# Effects of genotype and exercise on hepatic gene expression in phenotype-selected marathon mice

**DOI:** 10.1038/s41597-026-07935-4

**Published:** 2026-08-19

**Authors:** Anne-Marie Galow, Frieder Hadlich, Nares Trakooljul, Christina Walz, Andreas Hoeflich, Julia Brenmoehl

**Affiliations:** Research Institute for Farm Animal Biology (FBN), Wilhelm-Stahl-Allee 2, 18196 Dummerstorf, Germany

## Abstract

Experimental evolution through selection experiments is a vital tool for exploring the special features of polygenic traits. Here, we provide liver transcriptome data from a paternally selected marathon mouse model (DUhTP), characterized by exceptional running performance, and unselected controls (DUC), both descended from the same polygenic background. To study training responses, both lines were either challenged by three weeks of high-speed treadmill training or remained sedentary. For each condition, eight animals were used for liver tissue isolation and next-generation sequencing. Raw sequencing files were submitted to the ArrayExpress collection under accession number E-MTAB-12071. High data quality was confirmed by appropriate mean read lengths, high mean quality scores, and low duplication rates after adapter trimming. With an average of 16.9 million reads per sample mapping to 19,930 genes, the dataset enables comprehensive gene expression analyses. A multiQC report for the dataset, together with a sample-by-gene count matrix, is available on Figshare. The provided data offer a valuable resource for research in polygenic traits associated with physical activity.

## Background & Summary

Experimental evolution, including guided selection experiments, is a valuable tool for exploring the underlying genetic mechanisms of multiple traits^1^. At the Research Institute for Farm Animal Biology (FBN), the world’s longest selection experiment on mice began in 1969 from a founder line established from eight mouse strains. Systematic crossing of the inbred strains CBA/Bln, AB/Bln, C57BL/Bln, and XVII/Bln and the outbred strains NMRI orig., Han:NMRI, CFW, and CF1 gave rise to the founder line Fzt:DU (named after the institute, which used to be “Forschungszentrum für Tierproduktion Dummerstorf”)^2,3^. Starting from this breeding mouse strain, an unselected control line (DUC) was generated by random mating with up to 200 parent pairs per generation, as well as trait-selected mouse lines while avoiding inbreeding. One of these mouse lines was selected for high treadmill performance (DUhTP) over 140 generations, resulting in individuals with more than three times higher untrained running capacity than DUC mice^4,5^. Along with the enormous running capacity, increased lipid turnover has been observed in adipose tissue^6^, particularly subcutaneous fat^6,7^, as well as liver^5,8^ and muscle^9^.

Both lines have been comprehensively characterized at the genomic level^10^. Notably, DUC mice possess a broad genetic background, resulting in a much lower proportion of homozygous genome regions (Runs of Homozygosity (RoH) ≈25%) and a higher proportion of polymorphic alleles (≈75%) compared to DUhTP mice (RoH ≈60%, polymorphic alleles ≈40%)^10^. This genetic heterogeneity reflects the variability of human populations more adequately than inbred mouse strains, rendering DUC mice a more appropriate model for human physiology.

**Fig. 1:**
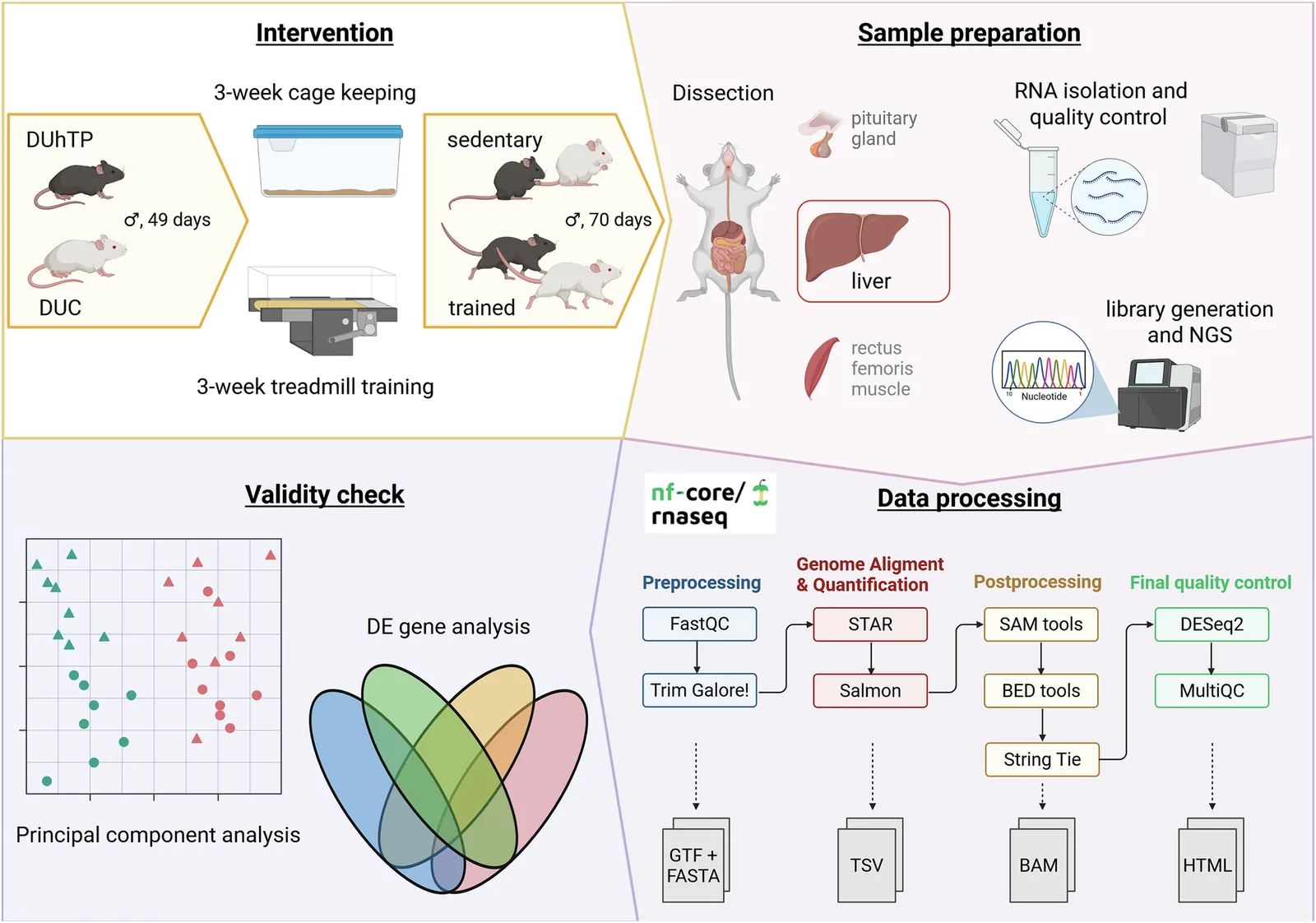
Illustration of the experimental design, including sample generation and data processing applying the nf-core/rnaseq pipeline.

Sequencing and differential gene expression analyses were performed on sedentary (sed) DUhTP and DUC mice, or on mice following three weeks of high-speed treadmill training (tr), to identify selection-driven adaptations in the liver, as presented here (Fig. 1) and other tissues^9,11,12^. The provided data were generated to investigate potentially distinct training-induced responses in the liver as part of this tissue crosstalk, offering a valuable resource for research in polygenic traits associated with physical activity. The raw sequencing files have been submitted to the ArrayExpress collection (https://www.ebi.ac.uk/biostudies/arrayexpress) under accession number E-MTAB-12071^13^, while a processed sample-by-gene count matrix has been made available at Figshare (10.6084/m9.figshare.29967283)^14^.

## Methods

### Animals and study design

The underlying experimental design of this study was described in previous publications^9,11,12^ but will be outlined in detail here for the purpose of comprehensibility. For this study, the long-term selected mouse line DUhTP and the unselected control line DUC were used. Both lines are non-inbred, originating from the same genetic background, an outbred line Fzt:DU. For the establishment of the DUhTP line, a single submaximal run on a computer-controlled treadmill was incorporated into the selection procedure for each generation, for four generations per year. For this, males completed a submaximal run after mating to determine the best runners and choose their offspring as the next generation’s parents, while avoiding inbreeding. So these DUhTP mice are characterized by an innate exceptional running ability, three to four times higher than the control line without prior training^4,5^, which is why the mice are also referred to as marathon mice. The males and females of the unselected control line DUC were randomly mated with up to 200 parent pairs per generation to represent the initial strain Fzt:DU.

The animal experiments were conducted at the FBN’s mouse facility in Dummerstorf, Germany. Our internal institutional review board approved all experiments, which complied with international and national animal welfare guidelines (Animal Welfare Act (TierSchG); AZ 7221.3.1.014/17). Consistent with the guidelines recommended by FELASA for hygiene management and health monitoring, male animals of generations 141 (DUhTP) and 185 (DUC) were housed in polysulfone cages measuring 267 × 207 × 140 mm (H-Temp PSU, Type II, Eurostandard Tecniplast, Hohenpeißenberg, Germany) under a 12-hour light-dark cycle (room temperature, 22.5 °C ± 0.2 °C; humidity, 45–60%) in the specific pathogen-free barrier unit. All animals had free access to water and autoclaved Ssniff^®^ M-Z food (61 kJ% carbohydrates, 27 kJ% protein, 12 kJ% fat; gross energy 16.7 MJ/kg; Ssniff-Spezialdiäten GmbH, Soest, Germany). After weaning, males from both lines were kept paired in a single cage until they were 48 days old. At that point, they were randomly split into two groups and housed in individual cages.

From 49 to 70 days of age, a subset of both mouse lines completed a 3-week exercise program (tr = “trained”, n = 8) on a computer-controlled treadmill (TSE Systems GmbH, Berlin, Germany), while the rest of the animals remained in their cages and served as a sedentary control group (sed, n = 8). Since the mice exhibit different running performances, we adjusted the training duration to match the running capacity of each strain, thereby challenging the animals equally. The basis for these treatments was the submaximal running times of earlier generations (DUhTP: 133 min, DUC: 66 min)^15^, with a training duration per session set at 30 minutes for DUhTP and 15 minutes for DUC, respectively. The training took place between 8:00 and 9:00 a.m. during daylight hours, five days a week, for three weeks. After an initial run and a two-day break, regular training (5 × per week, 15/30 min, 0% incline) began. The treadmill speed was initially 0.2 m/s for 20 s, then 0.36 m/s for 160 s, followed by a final speed phase. The final distance/speed was increased weekly until a final speed of 0.5 m/s was reached. Only the control mice were unable to meet the target speed of 0.5 m/s, so they completed the last week of training at 0.42 m/s. Before and after each run, mice were visually inspected to determine their body condition score. Additionally, the animals were weighed at days 49, 56, 63, and 70.

At 70 days of age, sedentary animals were sacrificed directly from their home cages, while the animals in the trained groups were sacrificed immediately after their final running session. In this way, we ensured that we could examine the impact of the current treadmill run within the context of a three-week training program. Mice were euthanized by rapid decapitation without anesthesia to prevent confounding effects of anesthetic agents, to avoid drug-induced metabolic and physiological artifacts, and to obtain chemically uncontaminated tissue samples. Blood samples were collected, and tissues (rectus femoris muscle, pituitary gland, epididymal fat, posterior subcutaneous fat, and liver) were isolated, weighed, shock-frozen in liquid nitrogen, and stored at −70 °C for subsequent analysis. Individual liver and body weights are presented in Table 1 and were analyzed for group differences using two-way ANOVAs with GraphPad Prism (version 10.6.1), which also identified the source of variation, reported to indicate the impact of the mouse line or treatment on the dataset’s variance.

**Table 1:** Individual weights of liver, carcass, and body in sedentary (sed) and trained (tr) DUhTP and DUC mice.

Sample	Strain	Condition	Body weight at day 49 [g]	Body weight at day 56 [g]	Body weight at day 63 [g]	Body weight at day 70 [g]	Carcass weight at day 70 [g]	Liver weight at day 70 [g]
*L-785*	DUhTP	tr	30.38	31.07	32.29	32.11	11.05	1.58
*L-788*	DUhTP	tr	31.79	31.09	32.10	31.88	10.82	1.48
*L-790*	DUhTP	tr	31.57	32.88	33.54	31.86	10.56	1.48
*L-792*	DUhTP	tr	33.82	35.41	35.07	34.36	11.84	1.77
*L-793*	DUhTP	tr	30.74	31.28	31.31	31.68	10.86	1.70
*L-794*	DUhTP	tr	28.35	28.13	28.85	30.06	10.30	1.72
*L-795*	DUhTP	tr	29.90	30.56	29.91	29.57	10.16	1.61
*L-796*	DUhTP	tr	29.49	28.92	30.24	28.59	9.50	1.44
*L-797*	DUhTP	sed	31.52	31.65	32.73	32.63	11.00	1.72
*L-798*	DUhTP	sed	33.81	34.99	34.34	36.67	12.42	1.93
*L-800*	DUhTP	sed	27.97	29.10	29.68	31.46	10.53	1.61
*L-802*	DUhTP	sed	29.05	29.27	30.05	31.84	11.07	1.50
*L-803*	DUhTP	sed	30.81	32.42	32.03	34.50	11.24	1.97
*L-804*	DUhTP	sed	31.89	31.71	31.80	34.20	12.03	1.88
*L-805*	DUhTP	sed	27.73	29.14	28.79	30.97	11.31	1.68
*L-806*	DUhTP	sed	31.15	31.69	32.66	34.42	11.20	1.76
*L-807*	DUC	sed	39.28	42.03	44.37	45.01	15.48	2.32
*L-808*	DUC	sed	37.82	39.30	40.65	40.71	14.02	2.22
*L-809*	DUC	sed	36.35	38.18	39.31	41.09	13.68	2.22
*L-811*	DUC	sed	34.08	35.45	37.27	38.72	14.52	1.94
*L-812*	DUC	sed	37.11	38.73	40.48	40.52	13.83	2.07
*L-813*	DUC	sed	37.63	39.63	41.49	43.13	14.78	2.38
*L-815*	DUC	sed	40.88	41.66	42.32	42.58	14.55	2.08
*L-816*	DUC	sed	34.33	35.56	36.61	37.05	13.06	1.82
*L-817*	DUC	tr	36.09	37.38	38.53	40.77	14.95	1.78
*L-818*	DUC	tr	36.99	37.62	38.46	37.64	14.19	1.84
*L-821*	DUC	tr	36.37	36.19	38.52	37.56	13.25	1.87
*L-823*	DUC	tr	34.39	36.46	36.91	37.85	12.77	1.95
*L-824*	DUC	tr	37.10	37.31	39.06	39.29	14.85	1.95
*L-826*	DUC	tr	34.55	34.75	36.23	36.80	13.66	1.88
*L-827*	DUC	tr	42.65	43.63	45.41	45.46	16.75	2.45
*L-828*	DUC	tr	37.40	37.94	38.24	38.94	13.79	2.04

### Library generation and sequencing

For RNA isolation, biopsies of the large liver lobe were dissected and homogenized in 1 mL of TRIzol^TM^ reagent (Invitrogen, Karlsruhe, Germany), before total RNA was extracted according to the manufacturer’s instructions and treated with DNase I using the RNase-free DNase kit (Base-Zero DNase, Biozym, Hessisch Oldendorf, Germany). Afterward, extracted RNA was enriched with the Zymo® RNA Clean&Concentrator kit (Zymo Research, Irvine, CA, USA). RNA quality was determined using an Agilent RNA 6000 Nano kit and an Agilent 2100 Bioanalyzer (Agilent, Waldbronn, Germany). Samples that passed quality control were used to generate a DNA library using a TruSeq Stranded mRNA Sample Preparation kit, following the manufacture’s protocol (Illumina, Berlin, Germany). Polyadenylated mRNA molecules were purified from 1 µg of total RNA using poly (T)-coated magnetic beads and chemically fragmented at elevated temperature. The fragmented RNA was then reverse-transcribed into cDNA using random hexamers and Superscript II reverse transcriptase (Invitrogen, Karlsruhe, Germany) and subsequently ligated to TruSeq RNA adapters containing a unique DNA sequencing index to facilitate multiplexing.

DNA library concentration was determined using a KAPA qPCR Library Quantification kit (KAPA-Biosystems, Wilmington, MA, USA) before libraries were quality controlled for fragment-size distribution using an Agilent Technologies 2100 Bioanalyzer and an Agilent DNA-1000 Chip kit. The normalized multiplexed DNA libraries with a 0.5% PhiX control were amplified and clonally clustered using the cBot system and then sequenced at 2 × 101 bp in high-performance mode on a HiSeq 2500 (Illumina, San Diego, CA, USA) at our sequencing facility at the FBN.

### Data processing and quality control

The raw sequencing reads (fastq) were quality-assessed using FastQC (version 0.12.1) and preprocessed with Trim Galore! (0.6.10)^16^, filtering out low-quality reads based on a mean Q-score < 30, read length shorter than 30 bp, and by removing adapter-like sequences. High-quality paired-end reads were then aligned to the Ensembl reference mouse genome GRCm39 and Ensembl reference mouse annotation version 114 using STAR (2.7.11b). Finally, Salmon (1.10.3) was used for gene quantification of transcriptome-based mapping results. All applied bioinformatic steps were accessible by the nf-core rnaSeq pipeline (3.19.0). Individual steps of the nf-core rnaSeq pipeline are illustrated in Fig. 1.

The resulting gene count data were used for PCA and differential gene expression analysis using the package DESeq 2 (version 1.52.0) within the R environment (version 4.6.0). First, Salmon’s gene count matrix was loaded along with a four-group design (DUC sed, DUC tr, DUhTP sed, DUhTP tr). Next, filtering was performed to retain only genes with at least 10 reads in at least six samples from each group to balance sensitivity against inclusion of low-confidence counts, resulting in 11,814 genes remaining. The DESeq 2 model internally corrects for library size, which eliminates the need for additional normalization before DESeq analysis. Significantly differentially expressed (DE) genes were selected based on a Benjamini-Hochberg-adjusted p-value below 0.05 and a log2 fold change greater than |1|. Finally, an Excel file was created containing tables listing the gene identification (Gen-ID), average counts, the fold change (log_2_FC), *p*-value, and false discovery rate for each comparison using the package writexl (version 1.5.4) (Supplemental Table 1).

The DE gene data obtained from the group comparisons were used to select genes that were regulated in dependence on the strain, i.e., genes that were significantly DE (adj. p < 0.05) in both sedentary DUhTP vs. DUC mice and trained DUhTP vs. DUC mice, or in dependence on the training, i.e., genes that were significantly DE (adj. p < 0.05) in both trained vs. sedentary DUhTP mice and trained vs. sedentary DUC mice, respectively. For these genes, a functional protein association network analysis was conducted using STRING^17^ (version 12.0). For GO “Biological process” analyses, we employed WebGestalt 2024^18^, applying the enrichment method GSEA and pathway_KEGG as enrichment categories. As this approach demands a minimum number of genes, all significant DE genes (adj. p < 0.05) were used as input for the GO analysis in each comparison. Thresholds for the number of IDs in a category were set to >5 and <5000. The number of permutations was set to 1000. Once the gene list (user IDs) of DE genes with FC had been loaded, the unique Entezgene IDs were assigned automatically by the program; however, a small proportion of the user IDs could not be functionally assigned and were therefore not included in the ‘Biological Process’ bar chart.

## Data Record

The provided dataset contains raw sequencing data from liver tissue of two mouse lines that underwent a 3-week exercise program, along with their corresponding sedentary control groups. The dataset has been submitted to the ArrayExpress collection (https://www.ebi.ac.uk/biostudies/arrayexpress) under accession number E-MTAB-12071, including an experimental description, sample metadata, and library preparation and sequencing protocols that have been uploaded to the BioStudies database (https://www.ebi.ac.uk/biostudies/)^13^. Additional data files comprise an Intermediate Data Format (IDF) file containing general metadata for the respective animal experiment and a Sample and Data Relationship Format (SDRF) file describing the relationship between the samples and the uploaded raw fastq files. Additionally, a multiQC report for the dataset together with a sample-by-gene count matrix is provided at Figshare (10.6084/m9.figshare.29967283)^14^.

## Data Overview

**Fig. 2:**
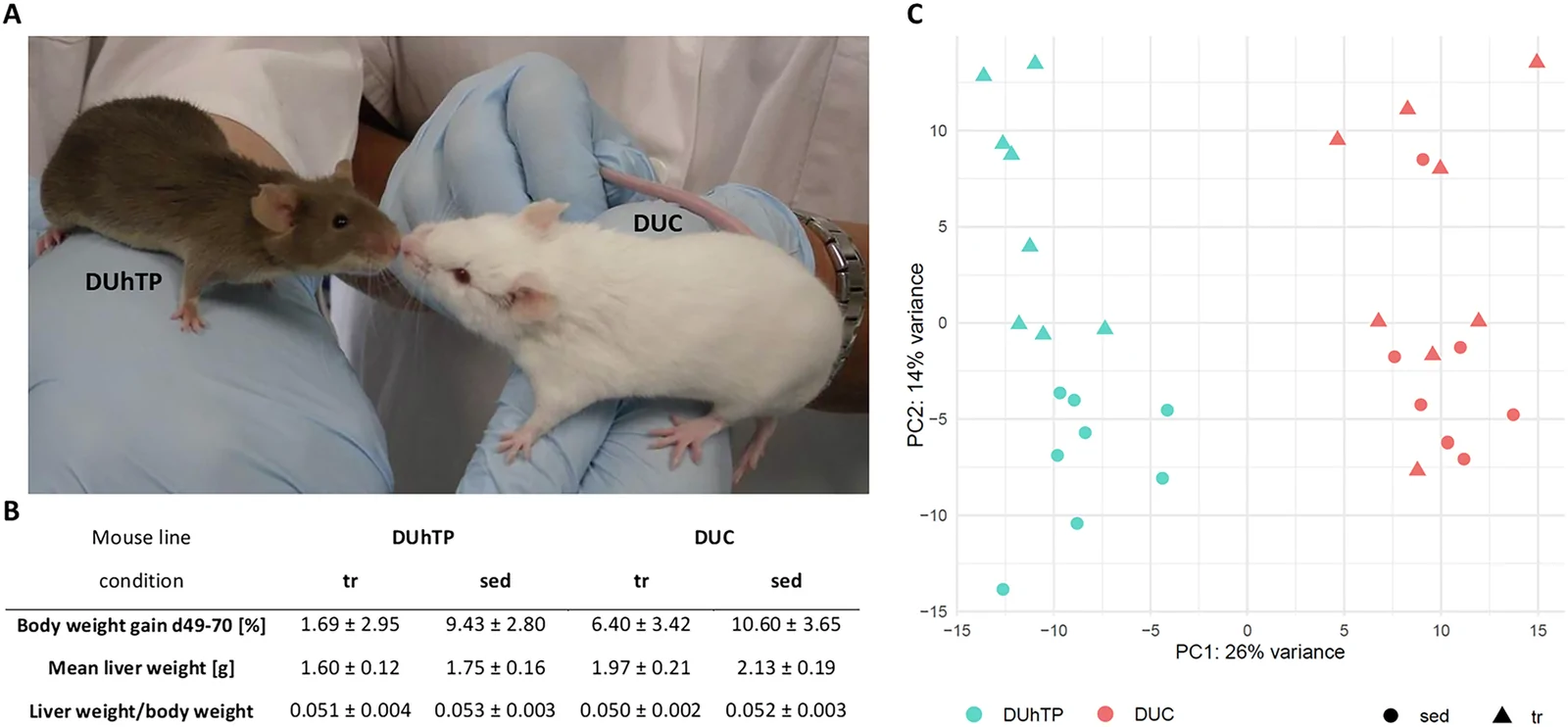
Overview of strain- and training-dependent features of DUhTP and DUC mice. (**A**) Phenotype of the mouse lines examined. (**B**) Weight parameters in trained and sedentary DUhTP and DUC mice based on the data given in Table 1. (**C**) Principal component analysis of DE genes performed with DESeq 2.

140 generations of selection led to changes in the phenotype of the DUhTP mice, including a reduction in overall size and total body weight (Fig. 2A), resulting in no significant differences in liver weight relative to total body weight between the groups (Fig. 2B). The lower liver weights in the DUhTP line, which are significant based on a two-way ANOVA (p < 0.0001), are attributed to lower body weights in the selection line compared to the control line (p < 0.0001) (Fig. 2A+B, Table 1). In both mouse lines, training significantly reduced liver weights (two-way ANOVA, p = 0.015), even though the training effect accounts for a much smaller proportion of the observed variance in the dataset than the impact of the mouse line (9.4% vs 51.6% of total variation). Notably, training also significantly affected body weight gain from day 49 to day 70 (two-way ANOVA p < 0.0001), with a more pronounced effect in DUhTP mice (Fig. 2B), indicating a genotype-dependent response to physical activity. Differences in gene expression were more apparent between the mouse lines than between the differential training statuses, as evident from the clear separation of the mouse lines in a PCA illustrated in Fig. 2C.

**Fig. 3:**
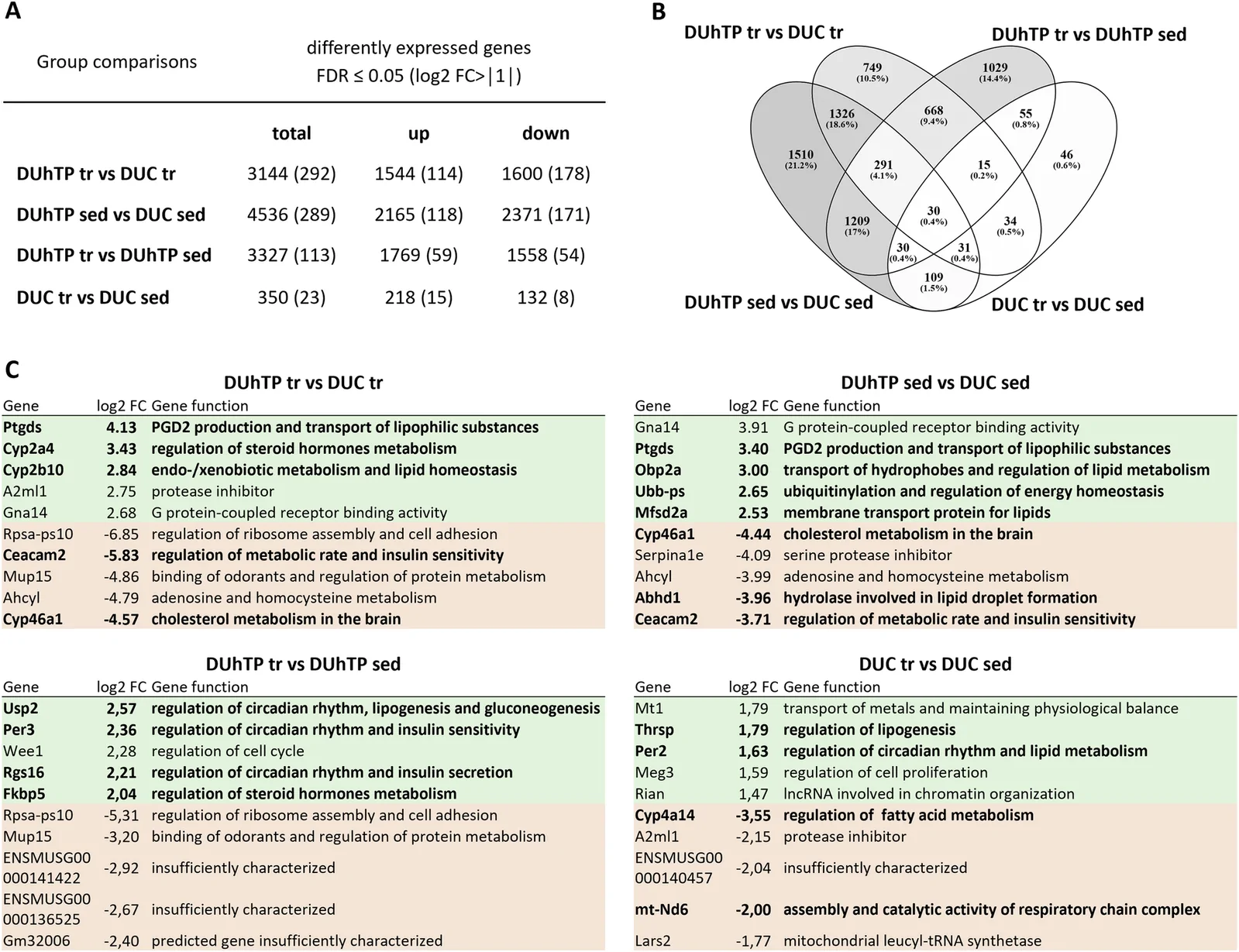
Results of differential gene expression analysis for relevant group comparisons. (**A**) Number of significant DE genes (FDR ≤ 0.05) for each of the four two-group comparisons. Numbers in brackets indicate strongly regulated genes with |log2 FC| > 1. (**B**) Venn diagram illustrating overlapping DE genes between group comparisons. (**C**) Top 5 up- and down-regulated genes and their function for all group comparisons. Genes involved in energy homeostasis and lipid metabolism are written in bold.

Comparisons between DUhTP and DUC mice yielded the most DE genes, while the fewest differences were observed between trained and untrained DUC mice (Fig. 3A,B). Overall, a high proportion of DE genes is involved in energy homeostasis and lipid metabolism (Fig. 3C), particularly in comparisons between DUhTP and DUC mice without training, but still notable in comparisons between trained and sedentary mice. This is in line with prior reports on metabolic adaptations in the liver and subcutaneous fat of this mouse line DUhTP^5–7^, thereby consolidating the validity of our presented gene expression data.

## Technical Validation

### Liver morphology

In all tested animals, the body condition score, ranging from 1 (emaciated) to 5 (obese), remained constant at a value of 3 throughout the experiment. Visual inspection of the isolated livers did not reveal apparent differences in morphology, neither between the mouse lines nor between trained and sedentary individuals. There was no evidence of fatty livers or other pathological conditions in any of the livers.

### Quality validation and analyses

**Fig. 4:**
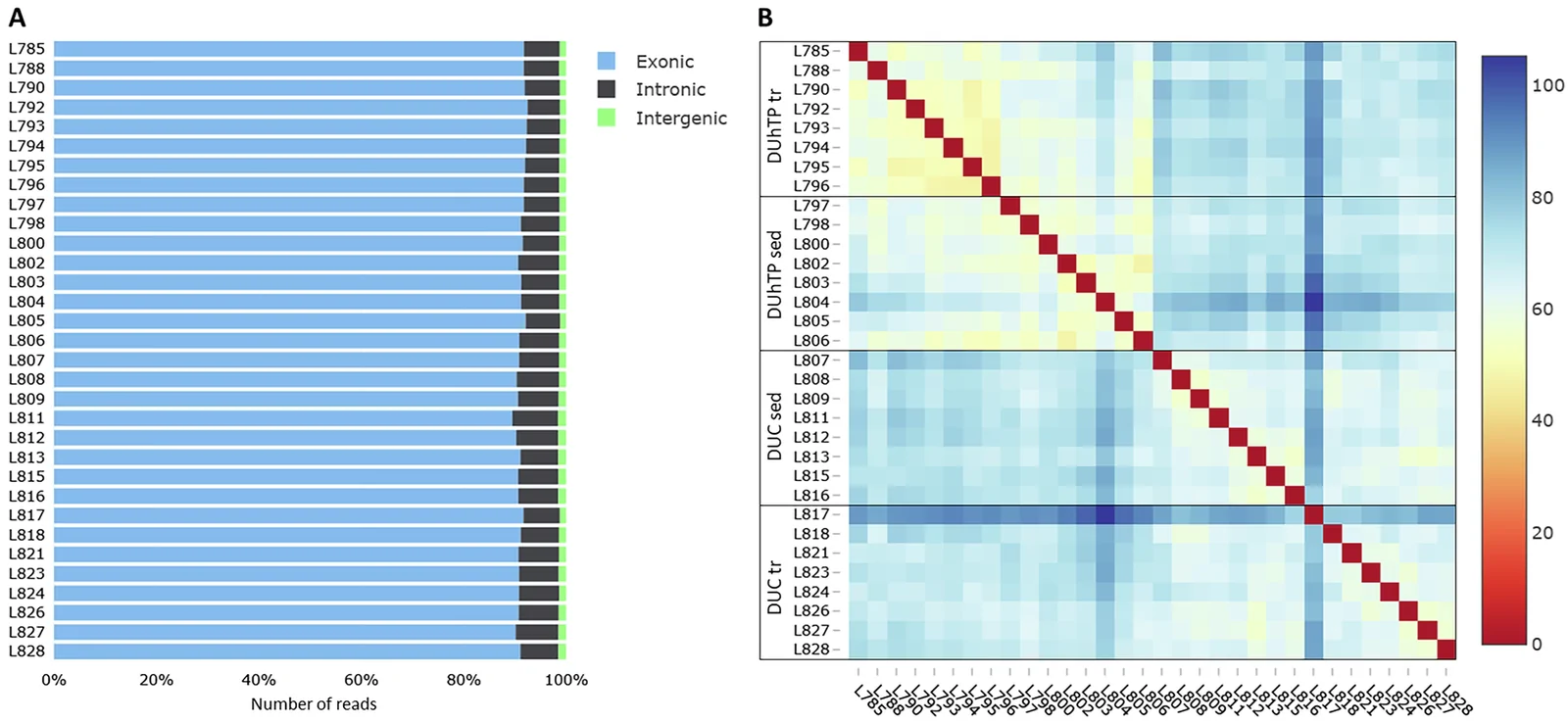
Assessment of data uniformity (**A**) Mapping results for all samples (% of reads mapping to introns, exons, intergenic regions, etc.) (**B**) Heatmap of sample-to-sample distances.

The integrity of the total RNA was measured using an Agilent 2100 bioanalyzer (Agilent, Waldbronn, Germany). All samples subjected to sequencing library construction had RNA Integrity Numbers (RINs) higher than 6 (7.14 ± 0.41). To validate overall sequencing quality, we analyzed parameters such as the sequence quality per base, GC content per sequence, sequence duplication levels, and quality score distributions across all sequences in the fastq files. Details for these and further measures can be explored for all samples via the multiQC report (v1.29) uploaded at Figshare (10.6084/m9.figshare.29967283). While ratios of reads mapping to introns, exons, and intergenic regions were similar in all samples (Fig. 4A), sample-to-sample distances generated with DESeq 2 demonstrated clear differences between the transcriptomic profiles of DUhTP and DUC mice (Fig. 4B). Notably, distances in the DUC groups were higher than in the DUhTP groups, with the highest sample-to-sample distance for sample 817 (trained DUC). However, quality control parameters did not stand out for any of the samples, indicating that differences in gene expression are not due to differences in library quality.

**Fig. 5:**
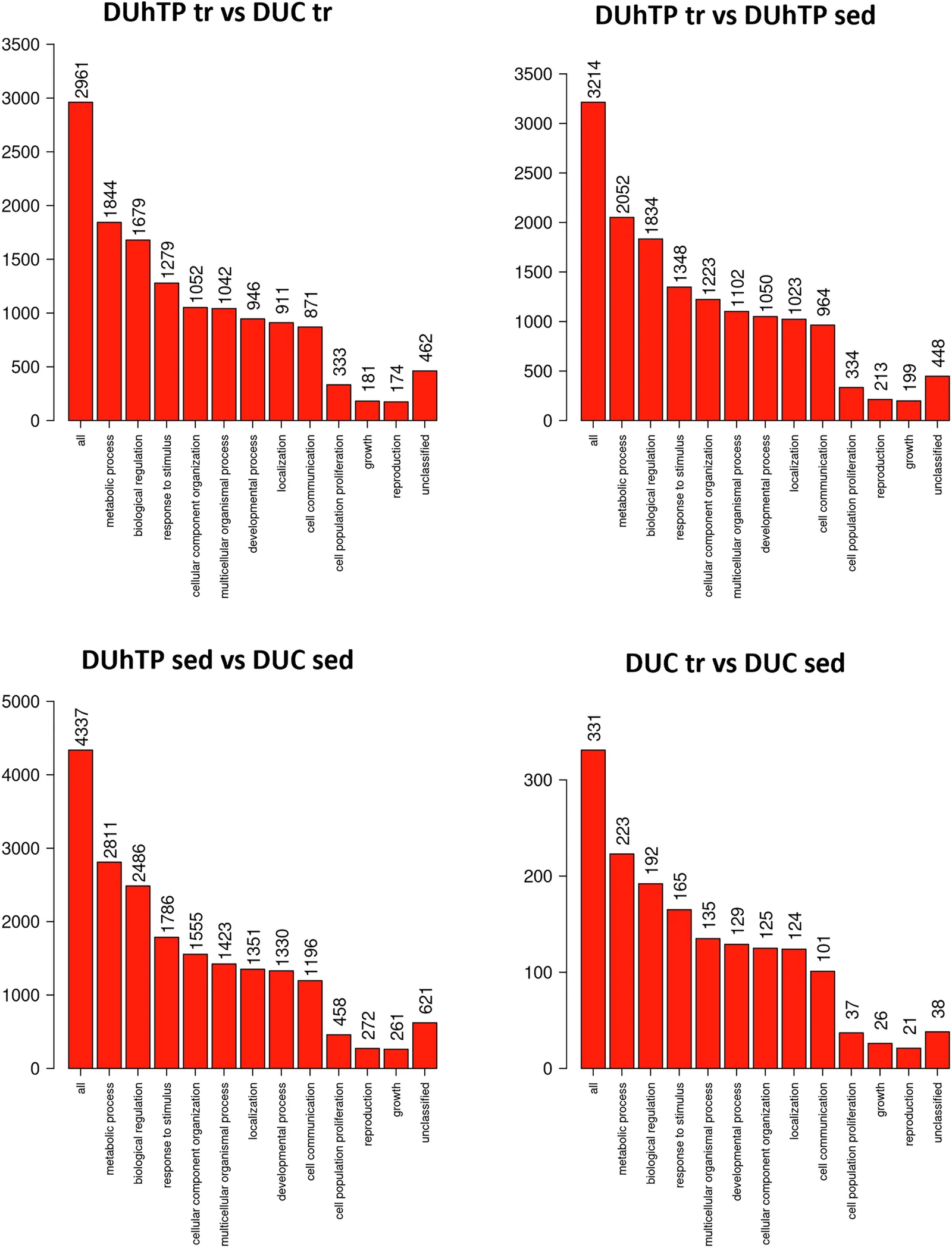
Biological process categories of all mapped IDs in the comparison groups of sedentary and trained DUhTP and DUC mice (DE genes defined by FDR < 0.05).

To obtain further indications of data integrity, the distribution of DE genes across the GO ontology category ‘Biological process’ was examined, which revealed that the majority of regulated genes was associated with metabolic processes for the comparisons of DUhTP mice with DUC mice (DUhTP vs DUC sed: 2811 of 4337 mapped DE genes, DUhTP vs DUC tr: 1844 of 2961 mapped DE genes) as well as for the comparisons of trained versus sedentary mice (DUhTP tr vs sed: 2052 of 3214 mapped DE genes, DUC tr vs sed: 223 of 331 mapped DE genes) (Fig. 5). Thus, the dataset is characterized by biologically plausible gene expression patterns and aligns with an earlier study using animals from both lines of the 95th generation, which used Affymetrix Mouse Array 430 A 2.0 data and a metabolic pathway analysis with AltAnalyze plus PathVisio^8^. In this prior analysis, comparing sedentary DUhTP mice with unselected controls, DE genes were enriched in several metabolic pathways identified by WikiPathway analysis (as demonstrated for cholesterol biosynthesis, steroid biosynthesis, fatty acid synthesis, glycolysis, and gluconeogenesis). Thus, the predominance of metabolism-related biological processes among DE genes, together with the agreement with previous transcriptomic analyses, further supports the integrity and reliability of the present dataset.

**Fig. 6:**
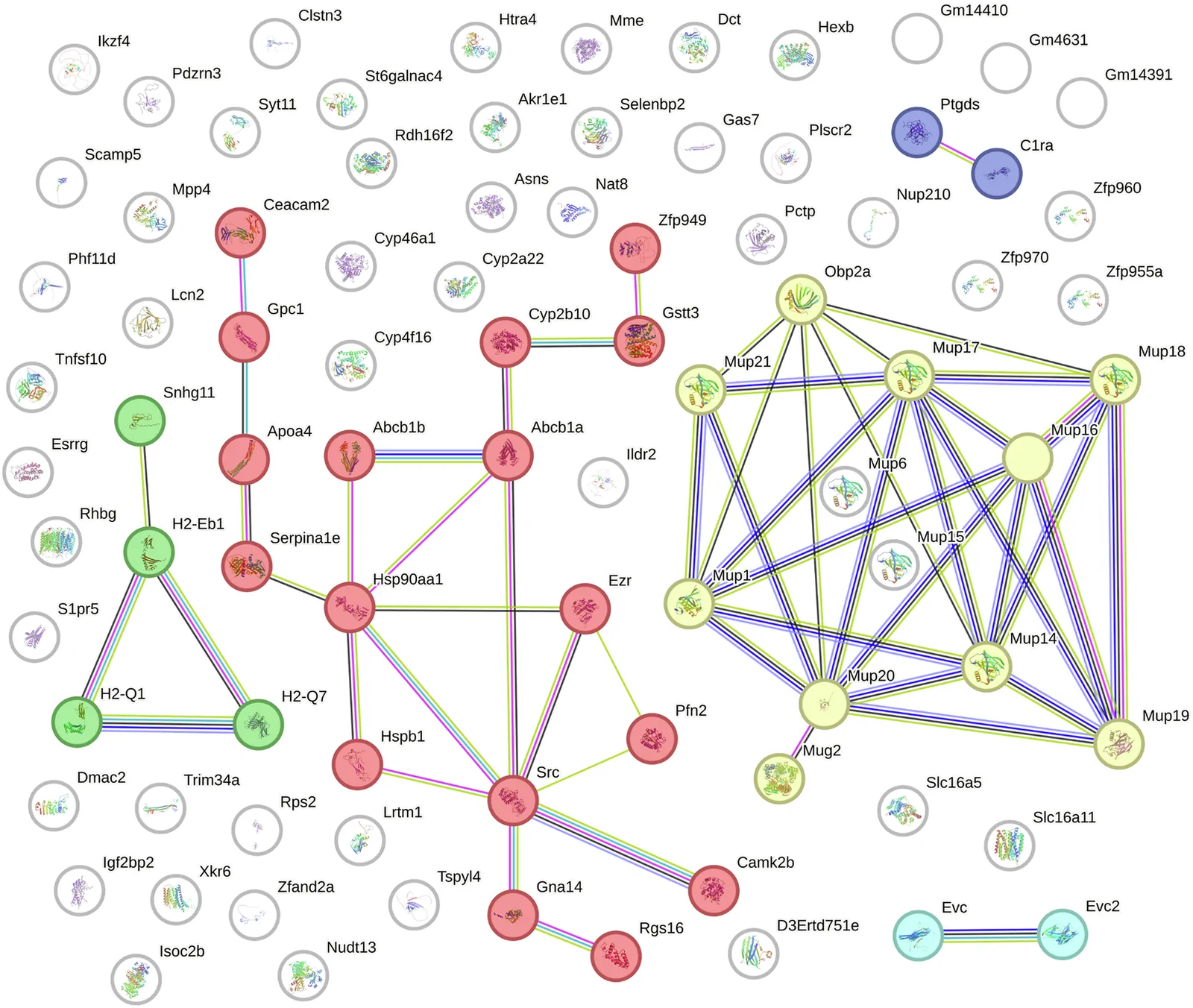
Protein association network for strain-dependent DE genes. Colored nodes represent query proteins and first shell of interactors, while white nodes represent the second shell of interactors. Colored lines define the type of interaction (cyan lines: known interactions from curated databases, purple lines: known interactions experimentally determined, dark blue lines: predicted interactions based on gene co-occurrence, light blue lines: protein homology, black lines: gene co-expression, golden lines: interaction based on text mining).

A functional protein association network analysis of genes regulated in both trained and sedentary DUhTP mice compared with the respective DUC group (Supplemental Table 1) showed a modulation of the Major Urinary Protein (MUP) family (Fig. 6). Since this family is implicated in energy expenditure and metabolism^19,20^, this finding consolidates the assessment of data integrity based on DE gene analysis.

With the chosen thresholds, seven genes were identified that were regulated in both DUhTP and DUC mice in response to training, forming no networks. Four of these genes (Per2, Thrsp, Eif4ebp3, and Fkbp5), which were all upregulated, are implicated in lipid metabolism and adipogenesis^21–24^, while three were yet uncharacterized (ENSMUSG00000140457 and Gm15889) or have a very basic function (Lars2 coding for a mitochondrial leucyl-tRNA synthetase), with ENSMUSG00000140457 and Lars2 being the only downregulated genes.

## Usage Notes

It should be noted that the animals in the training group (trained DUhTP and DUC mice) were euthanized immediately after the final treadmill run during the three-week training period; hence, the transcriptomic profiles reflect the combined state of three weeks of training and the last run. The mice in the control (sedentary) group were not subjected to any treadmill activity. When using the provided dataset to explore training effects, it might be worth considering the degree of heterogeneity, which can affect the number of significant DE genes, especially in the DUC mice. DUC mice exhibit a heterogeneous genetic background^10^, and sample-to-sample distances are higher in trained and sedentary DUC mice compared to DUhTP mice (Fig. 4B). Accordingly, adapted analytical strategies might be applied.

In particular, it is conceivable to divide the group of trained DUC mice into “responders” and “non-responders” based on the PCA results (Fig. 2C) in order to investigate differential responses to physical training, leveraging the individual-level phenotypic data provided in Table 1. Moreover, training in DUhTP mice upregulated several genes involved in circadian rhythm regulation, which suggests this mouse line as a model for investigations on the interplay of clock genes, energy homeostasis, and physiological traits.

Transcriptome data from the pituitary gland and the rectus femoris muscle, generated using the same experimental setup, are also available, providing datasets to shed light on crosstalk between the brain (pituitary gland), the metabolically active organ liver, and energy-consuming muscle tissue. Three publications present individual studies using these datasets to investigate energy metabolism and muscle protein metabolism^9,11,12^. The corresponding RNA-Seq data from the pituitary gland have been assigned ArrayExpress accession E-MTAB-12063^25^, and from the thigh muscle ArrayExpress accession E-MTAB-12072^26^ via https://www.ebi.ac.uk/arrayexpress.

## Supplementary information

Supplemental Table1

## Data Availability

The presented dataset is available as raw Fastq files and can be accessed via the ArrayExpress collection under accession number E-MTAB-12071, which also readily provides gene counts for all samples as plain text files. Additionally, a multiQC report for the dataset, together with a sample-by-gene count matrix is provided at Figshare (10.6084/m9.figshare.29967283). The full output of the conducted DE gene analysis is provided as a supplemental data table along with the manuscript, while the R script used for this analysis is provided at Figshare (10.6084/m9.figshare.29967283). Detailed information on *in vivo* experiments is provided in the manuscript in compliance with the ARRIVE guidelines 2.0.

## Code availability

All applied bioinformatic steps were accessible by the nf-core rnaSeq pipeline (3.19.0).

Trimgalore! (version 0.6.10): https://www.bioinformatics.babraham.ac.uk/projects/trim_galore/.

FastQC (version 0.12.1): https://www.bioinformatics.babraham.ac.uk/projects/fastqc/.

Ensembl reference mouse genome GRCm39: https://www.ensembl.org/Mus_musculus/Info/Index.

STAR (version 2.7.11b): https://github.com/alexdobin/STAR.

Salmon (version 1.10.3): https://github.com/COMBINE-lab/salmon.

R (version 4.6.0): https://cran.r-project.org/.

DEseq 2 (version 1.52.0): https://bioconductor.org/packages/release/bioc/html/DESeq2.html.
